# New Insights into Potential Beneficial Effects of Bioactive Compounds of Bee Products in Boosting Immunity to Fight COVID-19 Pandemic: Focus on Zinc and Polyphenols

**DOI:** 10.3390/nu14050942

**Published:** 2022-02-23

**Authors:** Meryem Bakour, Hassan Laaroussi, Driss Ousaaid, Asmae El Ghouizi, Imane Es-safi, Hamza Mechchate, Badiaa Lyoussi

**Affiliations:** 1 Laboratory of Natural Substances, Pharmacology, Environment, Modeling, Health, and Quality of Life (SNAMOPEQ), Faculty of Sciences Dhar El Mahraz, University Sidi Mohamed Ben Abdellah, Fez 30000, Morocco; meryem.bakour@usmba.ac.ma (M.B.); hassan.laaroussi@usmba.ac.ma (H.L.); driss.ousaaid@usmba.ac.ma (D.O.); asmae.elgouizi@usmba.ac.ma (A.E.G.); lyoussi@gmail.com (B.L.); 2 Laboratory of Inorganic Chemistry, Department of Chemistry, University of Helsinki, P.O. Box 55, FI-00014 Helsinki, Finland; imane.essafi1@usmba.ac.ma

**Keywords:** SARS-CoV-2, COVID-19, immunity, zinc, polyphenols, bee products, natural products

## Abstract

The coronavirus disease 2019 (COVID-19) is an epidemic caused by SARS-CoV-2 (severe acute respiratory syndrome coronavirus 2). Populations at risk as well as those who can develop serious complications are people with chronic diseases such as diabetes, hypertension, and the elderly. Severe symptoms of SARS-CoV-2 infection are associated with immune failure and dysfunction. The approach of strengthening immunity may be the right choice in order to save lives. This review aimed to provide an overview of current information revealing the importance of bee products in strengthening the immune system against COVID-19. We highlighted the immunomodulatory and the antiviral effects of zinc and polyphenols, which may actively contribute to improving symptoms and preventing complications caused by COVID-19 and can counteract viral infections. Thus, this review will pave the way for conducting advanced experimental research to evaluate zinc and polyphenols-rich bee products to prevent and reduce the severity of COVID-19 symptoms.

## 1. Introduction

On 11 March 2020, the World Health Organization recognized the new coronavirus disease 2019 (COVID-19) as a pandemic. The severe acute respiratory syndrome coronavirus 2 (SARS-CoV-2) was detected for the first time in Wuhan, China, at the end of 2019; the virus has not reported before in humans [1]. The virus is highly contagious and is transmitted through close contact with the contaminated person via respiratory droplets from coughing, and/or direct contact with contaminated surfaces [2]. Security measures imposed include washing hands frequently with soap and water, disinfecting them with a hydroalcoholic solution, wearing a mask when leaving the house, keeping to at least one meter away from anyone, avoid touching eyes, nose, or mouth when coughing or sneezing, and covering the nose and mouth with the bend of the elbow or with a tissue [3]. Most infected people have mild to moderate disease symptoms. The most frequent symptoms are fever, dry cough, fatigue, in addition to body aches, sore throat, diarrhea, conjunctivitis, headache, loss of smell or taste, rash, or discoloration of the fingers or toes; the most serious symptoms include difficulty in breathing or shortness of breath, feeling of tightness or pain in the chest, and loss of speech or motor skills [4,5]. Some infected people may be asymptomatic but they transmit the virus to other persons. Currently, there is no specific treatment for coronavirus infection. In non-hospitalized people, treatment aims to relieve symptoms and the most recommended active ingredient is paracetamol [6]. In other patients, doctors have introduced other drugs to treat the disease; these drugs were originally developed to treat other pathologies such as immunomodulatory drugs (e.g., interferons), and antiviral drugs which act by inhibiting the entry of the virus inside the cell (e.g., hydroxychloroquine, niclosamide, camostat), or by inhibiting viral proteases (e.g., lopinavir/ritonavir, PF-07321332, GC376, PF-07304814), other drugs inhibit viral RNA (e.g., remdesivir, molnupiravir), and finally the drugs that inhibit the host proteins which support viral protein synthesis (e.g., plitidepsin, fluvoxamine) [7]. Recently, Pfizer (New York, United States) has announced a new oral antiviral drug, ritonavir in combination with PF-07321332 (PAXLOVID™), that significantly reduces hospitalization and death by 89% of COVID-19 patients at high risk of severe disease [8,9]. The problem that still arises is the safety profile of these drugs. They require further evaluation such as evaluating the possible drug-drug interactions [10]. In addition, certain drugs are expensive and require intravenous administration supervised in a hospital [11].

The reason for the growing consumption of beehive products may be related to their health-oriented and therapeutic properties. The lookout for published studies on bee products and their ability to interact with SARS-CoV-2 and alleviate the symptoms of COVID-19 has attracted a lot of attention as they present a promising source of natural substances that can reduce severe symptoms of infected patients [12,13]. It is worth mentioning that several clinical trials have tested the combination between bee products and standard care to treat COVID-19 patients. For instance, in the trial number NCT04323345, there was a combination of honey given orally and through a nasogastric tube and standard drugs (lopinavir/ritonavir tablets or arbidol or chloroquine phosphate or hydroxychloroquine or oseltamivir with or without azithromycin) [14]. In addition, extract of standardized Brazilian green propolis, EPP-AF^®^ combined with standard care (azithromycin, chloroquine or hydroxychloroquine, oseltamivir, corticosteroids) were used for hospitalized COVID-19 patients in a trial registered under the number NCT04480593 [15]. The outcomes of these studies revealed that combining these natural products with standard care procedures has resulted in clinical benefits for hospitalized COVID-19 patients in comparison to the patients who only received standard care, such as the reduction in hospital stay length.

As there is currently no specific treatment against the COVID-19 pandemic, strengthening the immune system remains the right way to fight the disease. In this review, we suggest that bee products especially their richness in zinc and phenolic compounds may be a beneficial way to strengthen immunity and to protect against the virus SARS-CoV-2.

## 2. The Role of Immunity in Combating SARS-CoV-2

### 2.1. Mechanisms of the Immune Response against SARS-CoV-2

The immune system refers to our body’s global ability to resist and defend against different infections (fungi, protozoan, bacteria, and viruses). SARS-CoV-2 is one of these new pathogenic agents that triggers a coordinated immune response: innate immunity (rapid response), adaptive immunity (lower-acting), and passive immunity. When the human body encounters pathogenic antigens for the first time, in some people, especially vulnerable individuals the immune system cannot function quickly, complications may arise [16]. This has been observed in the case of COVID-19. The interface between innate and adaptive immunity immediately begins when the virus (SARS-CoV-2) reaches host cells [17]. This process goes through three essential steps: the identification of the spike glycoprotein of the virus, the elimination of the virus and the infected cells, and finally the development of immunological memory.

#### 2.1.1. The Identification of the Spike Glycoprotein of SARS-CoV-2

**Figure 1:**
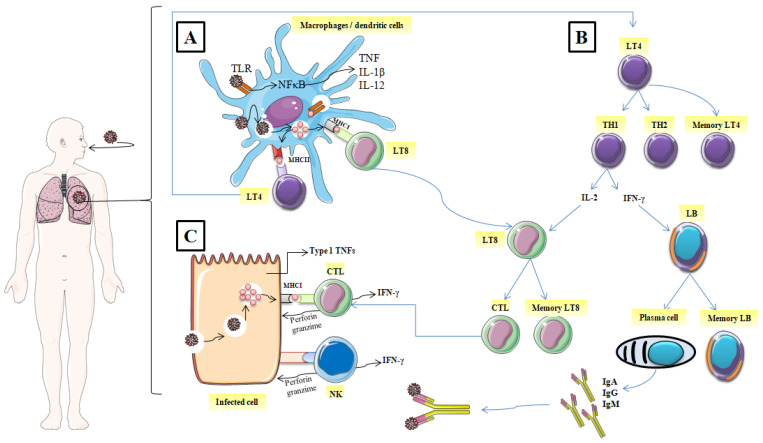
Mechanisms of the immune response against SARS-CoV-2. (**A**) identification of the spike glycoprotein of SARS-CoV-2, (**B**) activation of CD4^+^ helper T lymphocytes, (**C**) Elimination of the virus and the infected cell. (TLR: Toll-like receptors; NF-κB: nuclear factor-kappa B; TNF: tumor necrosis factor; IL: interleukin; MHCI: major histocompatibility class I; MCHII: major histocompatibility class I; LT4: CD4^+^ helper T lymphocyte; LT8: CD8^+^ helper T lymphocytes; TH1: T helper 1; TH2: T helper 2; IFNγ: interferon, LB: lymphocyte B; CTL: cytotoxic T lymphocyte; NK: natural killer; IgA: immunoglobulin A; IgG: immunoglobulin G; IgM: immunoglobulin M).

When the functional barrier of the immune system (respiratory tract and acid pH of the stomach etc.) fails to block SARS-CoV-2, monocytes, macrophages, and dendritic cells, antigen-presenting cells (APC) recognize and achieve it through the presence of pathogen recognition receptors (PRRs). Then, the first line of host defensive responses is activated. Owing to their intracellular Toll-like receptors (TLRs) especially, TLR3, TLR7, and TLR8, monocytes, macrophages, dendritic cells, and some other cell types recognize the single-stranded RNA of coronaviruses including SARS-CoV-2. Besides, extracellular PRRs identify the spike glycoprotein of the coronavirus coat (Figure 1A) [18,19].

#### 2.1.2. Elimination of the Virus, and the Infected Cell

Virally infected cells present on their surface virus antigenic determinants by major histocompatibility class I (MHC I), which are subsequently recognized by antigen-specific CD8^+^ cytotoxic T lymphocytes and then induce their restriction by the release of the effectors’ molecules such as perforin and granzymes (Figure 1C) [19]. Likewise, Natural killer cells (NK) also recognize and kill the infected host cells. The host immune cells adopted this strategy to slow down the virus invasion and thus, the interaction between the immune system and virally infected cells continues.

After the digestion of the internalized virus, termed antigens are recognized by specific TLRs and present on the surface of the innate immune cells (macrophage or dendritic cells) via MHC I (MHC I); [Human Leukocyte Antigen (HLA) in humans] and MHC II (HLA-Cw∗08) [20]. Furthermore, the recognition of antigen by PRRs enhances the expression of typical inflammatory cytokines such as IL-2, IL-18, IL-1β, type 1 IFNs (IFN-α and IFN-β), and tumor necrosis factor (TNF); it also activates inflammatory signaling and transcription factors such as inflammasome assembly and nuclear factor kappa-B cells (NFκB) [21]. In turn, these inflammatory cytokines initiate the activation of CD4^+^ helper T lymphocytes with the transition into a T helper (Th) which has a double function: cytokines produce and promote B Cells to generate antibodies. Being stimulated by intracellular pathogens, T helper lymphocytes 1 (Th1) phenotype promotes the cytotoxic T lymphocyte activity by releasing IL-2 and enhances the differentiation of B lymphocytes to plasma cells which produce specific antiviral antibodies by liberating IFN-γ (Figure 1B).

T helper lymphocytes (Th2) activate and promote the degranulation and the release of chemokines, proteases, and histamines by innate immune system cells including mast cells and basophils, which increased vascular permeability and enhanced the recruitment of macrophages and other inflammatory cells [22]. Besides, Th2 migrate to lung tissue and act by inducing airway hyper-responsiveness and metaplasia of the Goblet cells as documented in allergic illnesses [23]. Interactions (between TH1, TH2, and APC) are generally described as cellular cooperation. 

#### 2.1.3. Immunological Memory 

Thanks to immunological memory, the immune system can specifically recognize and immediately trigger an adequate immune response upon re-contact with an antigen previously encountered by the body (Figure 1B). In addition to the specific antibodies released into the circulation (IgM, IgA, and IgG), after the end of the active immune response, a pool of T memory cells are ready to fight against re-infection, leading to a fast elimination of the specific antigen source [24]. It has been observed that both CD8^+^ and CD4^+^ memory T cells in SARS-CoV-2 patients were effective in prompting a specific immune response from 3 months to 6 years without the presence of antigens [25]. Specific humoral immune response (anti-S-RBD IgM and anti-N IgG) against SARS-CoV-2 was found to have similar characteristics to that triggered against other coronavirus infections. IgG antibodies appear within 14 days of the onset of initial symptoms. However, IgA and IgM were detected on the fifth day after the first symptoms [26]. Positivity rates for IgM, IgA and IgG reached their maximum at weeks 4, 5 and 6, respectively [27].

## 3. Reasons for System Immune Failure

As an escape mechanism, SARS-CoV-2 uses different ways to beat the host immune system, lymphopenia and leukopenia being one of them [28]. After reaching the cell, SARS-CoV-2 encodes different proteins that interrupt molecules of JAKSTAT and TLRs signaling pathways. A recent study discovered that SARS-CoV-2 infection induced a high chemokine secretion and inhibits type I and type III interferon production which leads to the down expression of antiviral genes. Therefore, a decrease in the host’s immune response, especially T cells responses, is accompanied by hyper-inflammation [29]. Besides the reduction in helper T cells and suppressor T cells, SARS-CoV-2 prompts the dysfunction of effectors T cells. Persistent expression of inhibitory receptors such as programmed cell death 1 (PD-1), T cell immunoglobulin and mucin domain 3 (TIM-3), and T cell immunoglobulin and ITIM domain (TIGIT) in response to sustained antigen stimulation leads to T-cell exhaustion [30].

## 4. The Influence of Zinc on Immunity 

### 4.1. Importance of Zinc in Human Health

Zinc is a trace element that is neither synthesized nor stored by the human body, therefore it must be provided by the daily diet [31]. At a concentration of between 2 and 3 g, zinc represents the second most abundant trace metal in the body after iron, 90% of this concentration is found in muscle and bone; zinc is mainly concentrated inside cells and only 0.1% of the zinc concentration was found in plasma [32]. The cellular homeostasis of Zn is necessary for the proper functioning of the organism, the control of homeostasis is carried out by the importers of the ZIP family who are responsible for the transport of zinc in the cytosol, and the proteins of the ZnT family which export zinc out of the cytosol, and ultimately through zinc-binding proteins such as metallothionein [32].

Previous reports have shown the beneficial effects of zinc. It is very effective against anemia, it stimulates erythropoiesis [33], increases hemoglobin production [34], boosts antioxidant status, and protects against heavy metal toxicity [35]. Zinc supplementation in diabetic rats alleviates hyperglycemia, reduces protein glycosylation, and urinary concentration of proteins, urea, and glucose improves insulin resistance and the alteration of pancreatic morphology [36]. Concerning the endocrine system, Zinc plays an important role in the thyroid hormonal metabolism; its deficiency can slow down the activity of type I-5′deiodinase, an important enzyme that converts the hormone T4 into T3. It has been shown that zinc stimulates leptin secretion, it also acts on insulin and it participates in glycemic control [37]. A study conducted by Ko et al. [38] showed that zinc may increase the tolerance of chronic hepatitis C patients to the treatment with interferon and ribavirin.

### 4.2. Zinc, Immune System, and Coronavirus

Zinc is involved in the efficiency of the immune system; it acts as a modulator of the immune response via its essential role in the activation and maturation of B and T lymphocytes. Zinc deficiency has been shown to cause a decrease in the number of T and B lymphocytes in the thymus and bone marrow and leads to increased vulnerability to infections and weakened body defenses [39]. Among the arguments which prove the important role of zinc in the immune defense against SARS-CoV-2, one shows that it is deeply involved in the proper functioning of the immune system; for instance, recently it has been reported that zinc can block the interaction between the ACE2 receptor and SARS-CoV-2 spike proteins [40]. 

### 4.3. Effect of Zinc in Thymulin

**Figure 2:**
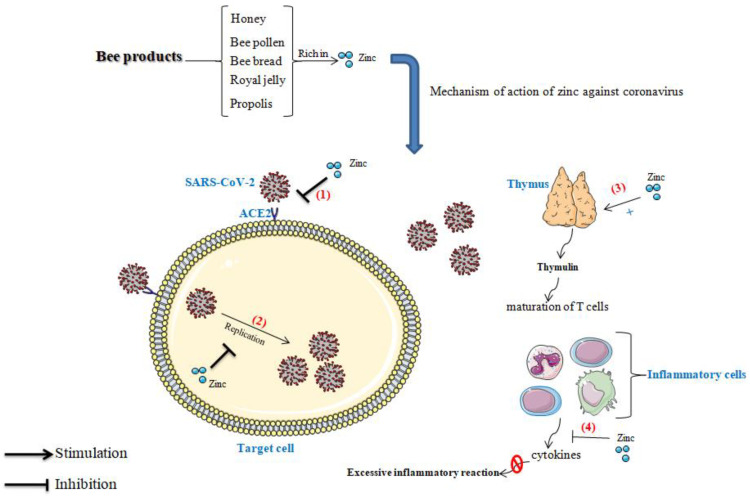
Role of zinc in immunity; (1) blocks the binding of the virus to the receptor; (2) blocks viral replication, (3): Active the thymulin function, (4) inhibit the excessive inflammatory reaction. (SARS-CoV-2: Severe Acute Respiratory Syndrome-Coronavirus 2; ACE2: Angiotensin-Converting Enzyme 2).

Thymulin or serum thymic factor is a nonapeptidic hormone produced by the thymus epithelial cells discovered in the 197. It has been shown that thymulin is involved in the differentiation of T lymphocytes, which are very important in the body’s defense against pathogens such as bacteria and viruses; thymulin is also an immunomodulator, it stimulates the immune system if it is weakened and it improves the actions of T cells and natural killer cells [41,42]. A study published by Nasseri et al. [43] revealed that thymulin can also alleviate inflammatory pain via the modulation of cell signaling pathways and molecular spine. Previous studies showed that the biological activity of thymulin significantly decreases during dietary zinc restriction (Figure 2), which proves that zinc contributes to the proper functioning of the immune system via its action on thymulin [41,44]. 

### 4.4. Role of Zinc in the Blockage of Viral Replication

Zinc is a very powerful antivirus agent; zinc deficiency has been reported to be strongly associated with viral infection risk [45]. Several studies have shown the inhibitory effect of zinc on viruses’ replication (Figure 2). Suara et al. [46] found in a study conducted in vitro that zinc salts blocked the replication of Respiratory Syncytial Virus, and prevented its cell-to-cell spread in HEp-2 cell monolayers pretreated with zinc, or when zinc was added after the infection to a semi-solid recovery medium. Similarly, it has been shown that zinc can reduce the replication of the hepatitis C virus (HCV), and thus, zinc supplementation may improve hepatitis in people with HCV infection [47,48]. In the same way, it has been reported that the replication of the hepatitis E virus was blocked via the inhibition of the activity of RNA-dependent RNA polymerase [49]. More importantly, zinc has shown a promising effect in Human Immunodeficiency Virus-1 (HIV-1) by inhibiting the viral transcription [50].

### 4.5. Preventive Effect of Zinc against the Excessive Inflammatory Reaction

Recent studies have shown that people who are infected with the novel COVID-19 present an aggressive inflammatory response with high levels of pro-inflammatory cytokines, which include interferon, interleukins, and chemokines. The cytokine storm is among the leading causes of death in the COVID-19 pandemic [51]. Molecularly, different signaling pathways have been involved in the inflammatory process; hence, the modulation of these routes is a major key to the management of the inflammatory response. The nuclear factor kappa-B (NF-κB) signaling pathway is one of them; it was found that zinc enhances the expression of peroxisome proliferator-activated receptor α (PRAR-α) and thus negatively regulates the NF-κB signaling pathway in the DNA nuclear, which leads to the minimization of adhesion molecules and pro-inflammatory cytokines generation [52]. Likewise, Haase et al. [53] documented that zinc plays its anti-inflammatory role by activating the A20 mRNA, the induction of protein A20 inhibits gene expression of TNF and IL-1-Induced NF-κB activation; and down-regulates Toll-like receptors (TLRs) pathway by removing K63-linked ubiquitin chains from the associated adaptor protein (Figure 2). 

## 5. The Antiviral Effect of Bee Products 

### 5.1. Propolis

Propolis is a sticky, lipophilic, resinous substance produced by a complex mixture of bee-released and plant-derived compounds. It is applied by honeybees as a hive defensive material against various infections; it is generally made up of around 50% resin, 30% waxes, 10% essential oils, 5% pollen, and 5% of various organic compounds [54,55,56]. The antiviral effect of propolis is well documented, it was reported that propolis samples from Brazil, Canada, Czech Republic, France, Italy, and China has promising antiviral efficacy against several viral infections such as herpes simplex virus (HSV) types 1 and 2, poliovirus type 1 (PV1), poliovirus type 2 (PV), vesicular stomatitis virus (VSV), adenovirus type 2 (Adeno-2), varicella-zoster virus (VZV), Human immunodeficiency virus type 1 (HIV-1), and influenza A virus H1N1. The virucidal effect of propolis against these viruses was presented through multiple mechanisms, including the induction of virion damage, the inhibition of viral compounds necessary for adsorption or entry into host cells, the degradation of RNA before virus entry into cells, the inhibition of viral growth, and the affection of the replication steps of the viral cycle in cells [57,58,59,60,61,62,63,64]. 

During this pandemic, propolis has become the object of many scientific investigations, such as a recent study published by Miryan et al. [65]. The study was carried out on 80 people aged between 18 and 75 years who had tested positive for SARS-CoV-2 by the PCR technique; the study was based on the supplementation of half of the patients (*n* = 40) with the propolis tablet at a rate of 300 mg 3 times per day for 2 weeks and the other half (*n* = 40) by a placebo tablet in order to assess the effect of propolis on the clinical symptoms caused by the SARS-CoV-2. The results obtained showed that the propolis improved the clinical symptoms and decreased the duration of the disease. Similarly, in a randomized controlled trial conducted by Silveira et al. [15], hospitalized COVID-19 patients were allocated to receive standard care plus an oral dose of 400 mg or 800 mg/day of Brazilian green propolis for 7 days, or standard care alone; then, the patients were followed for 28 days after admission. The results of this study showed that the length of hospital stay post-intervention was shorter in both propolis groups than in the control group. In addition, in the group of patients who received 800 mg/day of propolis, there was a lower rate of acute kidney injury than in the controls. Additionally, in a monocentric, randomized, double-blind, placebo-controlled clinical trial conducted by Esposito et al. [66], the results showed that the patients who suffered from symptoms of mild upper-respiratory tract infection (sore throat, muffled dysphonia, and swelling and redness of the throat) recovered after five days of oral spray supplementation of a polyphenol mixture extracted from poplar-type propolis. In a randomized clinical study published by Kosari et al. [67], the results revealed the efficacy of a syrup containing propolis and *Hyoscyamus niger* L. extract in the reduction in the COVID-19 symptoms. Likewise, the results of Bilir et al. [68] showed that the supplementation of healthcare professionals with Anatolian propolis can significantly protect them (98% of participants) against COVID-19 infection. 

Refaat et al. [69] developed an optimized liposomal formulation for the effective delivery of propolis components, enhancing their anti-viral activity against COVID-19. The results of molecular docking have shown that all of the propolis components have a high binding affinity to COVID 3-CL protease and spike protein compared to standard antivirals (avigan, hydroxychloroquine, and remdesivir). In addition, the docking analyses showed that octatriacontyl pentafluoropropionate present in Egyptian propolis has an excellent binding manner with the active site of RNA-dependent RNA polymerase, spike protein S1, and main protease [70].

### 5.2. Royal Jelly 

Royal jelly is a yellow-white creamy substance secreted by the mandibular and hypopharyngeal glands of young-worker bees [71]. The antiviral effect of royal jelly has been proved against many viruses such as hepatitis C virus and hepatitis B virus; it has been found in vitro that major royal-jelly protein (MRJP) 2 and its isoform X1 have proved their ability to inhibit the viral receptors CD81 and scavenger receptor class B type I (SR-B1), and also, they targeted HCVNS3/NS4A protease, HCV-NS5B polymerase and HBV polymerase (DNA-dependent DNA polymerase and reverse transcriptase) [72]. In addition, Hashemipour et al. [73] studied the effect of royal jelly on herpes simplex virus type 1 (HSV-1) and found that can inhibit the virus at a concentration of 250 μg/mL and can decrease the viral load from 70,795 to 30 PFU/mL at a concentration of 100 μg/mL. Importantly, in silico analyses revealed that (MRJP) 2 and the isoform X1 MRJP2 may be a promising therapy against SARS-CoV-2. These proteins can block the virus invasion and curb its replication, as well as they can hydrolyze sialic acid from the surface of lung cells [74]. In a randomized controlled trial, patients affected by SARS-CoV-2 have orally received three bee products including N-chromosome royal jelly, propolis and honey. At the end of the study, the results revealed that the total symptoms duration and the time to return to work were significantly reduced in the intervention group in comparison to the control group [75].

Furthermore, it was found that the major royal jelly glycoprotein, apalbunin1, is an immunostimulator compound and exert proinflammatory activities, by upregulating the production of tumor necrosis factor-a (TNF-a) [76]. In addition, in a study conducted by Yang and coworkers, the production of pro-inflammatory cytokines, Interleukin (IL)-8, IL-1β, and tumor necrosis factor-alpha (TNF-α) in WiDr cells was significantly modulated by 10-hydroxy-2-decenoic acid, the major fatty acid present in royal jelly [77]. Similarly, Kohno et al. showed that royal jelly has anti-inflammatory effectsthrough inhibiting proinflammatory cytokine production by activated macrophages stimulated with lipopolysaccharide or with lipopolysaccharide plus IFN-γ [78].

### 5.3. Honey 

Honey is a sweet substance made by bees, composed mainly of carbohydrates (60–85%) and water (12–23%). It also contains other compounds such as minerals, amino acids, vitamins, organic acids, and phenolic compounds [79]. Since archaic times, honey has been used in traditional medicine to treat several diseases, and it is known for its antioxidant, antimicrobial, wound healing, anticancer, and anti-diabetic effects [80,81]. The antiviral property of honey has been shown against many viruses. For instance, in a study conducted by Shahzad et al., the results revealed that manuka and clover honeys were effective against the varicella-zoster virus with an approximate EC_50_ = 4.5% (wt/vol) [82]. Similarly, Watanabe et al. have proved, in vitro, the capacity of manuka honey in inhibiting the influenza virus H_1_N_1_ [83]. These studies lead scientists to think about this hive product to test it against the new coronavirus (SARS-CoV-2). In a randomized controlled trial, the authors showed the prophylactic potential of honey against the hospital and community-based SARS-CoV-2 spread [84]. 

El Sayed et al. [85] were studied the effect of TaibUVID nutritional supplements (*Nigella sativa*, chamomile, and natural honey) as adjutants for COVID-19 contacts, patients, and public prophylaxis. The results obtained revealed that 70% of COVID-19 contacts (*n* = 14) (on regular TaibUVID intake) did not develop SARS-CoV-2 infection, while, 30% of COVID-19 contacts (*n* = 6) were not regularly using TaibUVID and developed mild flu-like symptoms. In addition, in the group of COVID-19 patients, 70% were (*n* = 14) improved in 1–4 days, 25% (*n* = 5) were improved in 5–10 days while 5% were improved in more than 10 days. Mustafa et al. [86] proposed that the anti-inflammatory properties of honey may reduce the severity of pulmonary manifestations in COVID-19 infections. Importantly, in a clinical trial, the efficacy of natural honey in the treatment of 1000 participants patients infected with COVID-19 in comparison with standard care was studied. The primary results showed that honey can reduce the recovery period to fourteen days [14].

### 5.4. Bee Pollen, Bee Bread, and Bee Venom

To produce bee pollen, the bees collect the pollen grains from the flowers mix them with the nectar and their enzymes, transport them to the hive in the form of pellets, then store them in the cells of the hive which are then sealed with wax. After that, lactic fermentation is carried out to form what is called bee bread [87]. It has been shown that bee pollen and bee bread from different botanical sources exert significant antiviral activity against the EV-D68 virus, with IC_50_ values ranging from 0.048 to 5.45 mg/mL [88].

The bioactive compounds of Korean *Papaver rhoeas* bee pollen (kaempferol-3-neohesperidoside, kaempferol-3-sambubioside, kaempferol-3-glucoside, quercetin-3-sophoroside, luteolin, and chelianthifoline) were tested against H_1_N_1_, H_3_N_2_, and H_5_N_1_ viruses. The results showed that all of the compounds have an antiviral activity with IC_50_ values ranging from 10.7 to 151.1 µM [89]. In a randomized controlled trial, the use of bee pollen as supplementation for COVID-19 patients significantly reduced the total symptoms duration and the time it took to return to work [75].

Bee venom is a colorless liquid produced by specialized caudal glands possessed by worker bees [90]. It has been used as a therapeutic modality since ancient times in Eastern Asia [91]. Bee venom is cytotoxic at a high dose; however, despite this toxicity, low doses of this hive product ranging from 1 to 3 μg/mL can be effective in the treatment of many illnesses such as rheumatoid arthritis, diabetes, and inflammation [92]. The bee venom contains many bioactive compounds such as volatile molecules, enzymes, and peptides including melittin, apamin, mast cell degranulating peptide, and adolapin [90]. The viricidal property of bee venom and its active compounds “melittin” were shown against several enveloped and non-enveloped viruses in vitro including respiratory syncytial virus, influenza A virus, vesicular stomatitis virus, enterovirus-71, coxsackie virus, and herpes simplex virus, and against H_1_N_1_ in the mouse model in vivo, and it has been confirmed that the antiviral mechanism of bee venom was the direct interaction with the viral surface, it stimulate type I IFN and inhibit viral replication [93]. 

In a survey study, 5115 beekeepers were surveyed, of which 723 were in Wuhan, China. It was found that none of the beekeepers developed symptoms associated with COVID-19. In addition, in an apitherapy clinic, the authors followed 121 patients who received bee venom from the honeybee’s sting to treat or prevent certain diseases. The follow-up of these patients revealed that none of them were infected by SARS-CoV-2, even those who were exposed to suspected infected cases [94].

#### Concern of Safety of Bee Products

Questions can be raised about the safety of some bee products such as bee pollen, bee bread, and bee venom. They can cause side effects and allergic reactions in certain people, such as local anaphylactic skin reactions, giant dermatofibroma with granular cell changes, and in some serious cases, they can cause dangerous anaphylactic reactions [95,96,97]. Therefore, special attention should be paid to avoid the incidence of adverse effects when using these products.

The allergic effect of bee venom is due to its histamine and enzymes, mainly hyaluronidase and phospholipase A2 [95,98], while the allergenic substances in bee pollen and bee bread are the pollen of anemophilous plants, and fungi such as Alternaria and Aspergillus which contaminate bee pollen and can induce allergic reactions after its ingestion [99]. Removing allergenic substances from bee products may be the right solution for avoiding any side effects. This has already been proposed by Aliboni et al. [100], who used a natural lipidic phase (bee wax + corn oil) to remove a high percentage of allergens from propolis.

However, many studies recommend bee pollen and bee venom therapy. This is due to their noteworthy pharmacological effects. It has been proved that bee venom acupuncture is effective in musculoskeletal pain, neuropathic pain, neuropsychiatric disorders, immunological diseases, rheumatoid arthritis, Parkinson’s, and skin diseases [101,102]. Similarly, it has been documented that bee pollen and bee bread can regulate ovarian functions, prevent obesity, and ameliorate blood sugar levels [103,104].

Conversely, it was revealed in a preclinical study that bee pollen phenolic extract had an anti-allergic effect via the decrease in IgE and IgG production, as well as the inhibition of the paw edema, the migration of leukocyte to bronchoalveolar lavage; it was also demonstrated the partial protection on the anaphylactic shock reaction induced in an experimental model of allergy (ovalbumin). The authors revealed that myricetin (a flavonoid compound that existed in the bee pollen) is responsible for the anti-allergic effect observed by bee pollen extract [105]. In addition, the anti-allergic property of bee venom was demonstrated in vivo, and the results revealed that bee venom can attenuate goblet cell hyperplasia and inflammatory cell infiltration, inhibit the production of IgE and IL-4, and mitigate nasal symptoms [106]. Chung et al. [107] also proved that bee venom has anti-allergic potential and anti-inflammatory properties via the regulation of the signal transducers of transcription 1/interferon regulatory factor 3 (STAT1/IRF3) signaling pathway and the reduction of inflammatory mediator production (prostaglandin E2, TNF-α, and IL-1β).

Several published reports support the use of these bee products to fight SARS-CoV-2, the main argument which is proposed is that allergens cause an elevation of specific IgE and IgG antibodies. They may provide advantages against SARS-CoV-2 because these IgEs can respond to a variety of antigens [92,108]. Similarly, other investigations showed that these bee products are potential adjuvants against COVID-19 by dint of their immunomodulatory and anti-inflammatory effects. It was proven that bee venom can modulate the immune system [101]. The phospholipase A2 (PLA2), an active component of bee venom belonging to the group III sPLA2, can act also as a ligand for specific receptors. It binds to the CD206 of dendritic cells and causes the secretion of prostaglandin E2 leading to the differentiation of CD4^+^CD25^+^ regulatory T cell (Treg) [109]. Later, these are important actors in the prevention of autoimmune diseases, acting by limiting the inflammatory reaction [110]. 

Concerning bee pollen, it was found that zinc-rich polysaccharides isolated from bee pollen exhibited an immunomodulatory property via the increase in nitric oxide secretion, interleukin-1β, tumor necrosis factor-α, and interleukin-6. These polysaccharides, in addition to their high levels of zinc content, are composed of glucose, arabinose, galactose, mannose, rhamnose, xylose, and galacturonic acid [111]. In the same way, it was shown that water-soluble polysaccharides isolated from bee pollen of *Crataegus pinnatifida Bge* possessed good immunostimulatory activity; they stimulated spleen cell proliferation and enhanced the phagocytic rates and phagocytic indexes of macrophage [112]. Additionally, in a study conducted by Shaldoum et al. [113], the results showed that bee pollen maintains the structure of bone marrow and spleen tissue, reduces apoptosis in hematopoietic cells, increases the production of hematopoietic stimulating cytokines-growth factors.

However, before the recommended bee products are used as complementary medicines against COVID-19, well-designed clinical trials are needed.

## 6. Role of Bioactive Compounds in Bee Products in the Management of COVID-19

### 6.1. Zinc

#### 6.1.1. The Content of Zinc in Bee Products 

In addition to the several bioactive compounds presented in bee products, mineral elements present important ingredients; they are used as a marker of their quality, safety, botanical, and geographical origin [114]. Additionally, bee products present a good source of zinc, and its role as supplementary of the deficiencies cannot be overestimated. Hence, considering the importance of zinc in the treatment of COVID-19 [115,116], bee products rich in zinc could improve symptoms caused by SARS-CoV-2.

Several studies were designed to determine zinc content in beehive products from different countries. As presented in Table 1, the Moroccan bee bread contains 3.31 ± 0.4 mg/kg of zinc, the Romanian samples have a content ranging between 29.54 and 31.85 mg/kg, the Malaysian bee bread sample contains 60.6175 ± 11.8112 mg/kg, the Lithuanian bee bread contains from 11.5 to 42.7 mg/kg of zinc, and the Turkish samples contain values oscillating between 52.55 and 73.96 mg/kg [87,117,118,119,120]. On the other hand, zinc content in bee pollen samples harvested from different geographical locations in Turkey was ranged from 14.832 to 68 mg/kg [87,121,122,123]. Indeed, in Brazil, the studies showed that the zinc content ranged from 39 to 76 mg/kg in bee pollen. Furthermore, other investigations reported that bee pollen samples from Serbia, Spain, Jordan, Slovakia, Poland, China, and Greece showed high contents of zinc, ranging from 18.8 to 159.3 mg/kg [124,125,126,127,128,129,130].

Propolis is another bee product very rich in mineral elements, especially zinc. According to the studies carried out on propolis samples originating from Argentina, Turkey, Poland, Spain, Brazil, Chile, Lithuania, Serbia, Greece, the content in Zinc ranged from 5.5 to 460.7 mg/kg [120,131,132,133,134,135,136,137,138,139,140]. Honey is the most studied bee product for mineral content, and zinc has been analyzed by several researchers [141,142,143,144,145,146,147,148,149,150,151,152,153,154,155]. The lowest contents were found in honey from Kenya, where the values ranged between 0.05 and 0.35 mg/kg. However, the highest values were recorded for honey from Malaysia (from 4.70 to 173.77 mg/kg).

Concerning royal jelly, samples produced in France, China, Germany, and Lithuania were analyzed and showed a content of zinc ranging from 17.56 to 26 mg/kg [120,156,157,158].

**Table 1:** The zinc content of bee products (bee bread, bee pollen, propolis, royal jelly) from different countries.

Bee Products	Country	Collection Period	Content in Zinc	Reference
Bee bread	Lithuania	2018	From 11.5 to 42.7 mg/kg	[120]
	Morocco	-	3.31 ± 0.4 mg/kg	[117]
	Romania	-	From 29.54 to 31.85 mg/kg	[119]
	Malaysia	-	60.61 ± 11.81 mg/kg	[118]
	Turkey	April to September 2018	From 52.55 to 73.96 mg/kg	[87]
Bee pollen	Italy	2018	25.5 mg/kg	[120]
	Denmark	2018	22.3 mg/kg	[120]
	Sweden	2018	23.3 mg/kg	[120]
	Slovakia	2018	28.7 mg/kg	[120]
	Poland	2018	31.7 mg/kg	[120]
	Lithuania	2018	From 20.3 to 27.8 mg/kg	[120]
	Ukraine	2018	From 20.8 to 22.1 mg/kg	[120]
	Latvia	2018	From 23.5 to 24.7 mg/kg	[120]
	Turkey	(April to September 2018)	From 39.37 to 68 mg/kg	[87]
	Serbia	(spring and summer of 2011)	From 28.76 to 75.92 mg/kg	[159]
	Turkey	-	From 25.94 to 49.74 mg/kg	[121]
	Brazil	August 2005 to April 2006	From 45.07 to 55.22 mg/kg	[160]
	Spain	1993	From 18.8 to 81.1 mg/kg	[129]
	Jordan	March to October 2017	From 25.24 to 77.02 mg/kg	[124]
	Turkey	-	From 14.832 to 39.037 mg/kg	[122]
	Slovakia	Spring season 2007	From 31.9 to 39.9 mg/kg	[128]
	Poland	June or August 2009.	From 75.2 to 159.3 mg/kg	[125]
	China	Flower season of 2010	From 28.25 to 65.30 mg/kg	[130]
	Turkey	-	From 20.21 to 59.57 mg/kg	[123]
	Brazil	-	From 39 to 76 mg/kg	[161]
	Greece	March to October 2018	From 24 to 90 mg/kg	[127]
Propolis	Poland	2018	52.4 mg/kg	[120]
	Lithuania	2018	From 31.9 to 102.1 mg/kg	[120]
	Argentina	-	From 53 to 68 mg/kg	[136]
	Turkey	-	From 17.60 to 67.60 mg/kg	[133]
	Poland	-	From 16.88 to 99.68 mg/kg	[138]
	Spain	-	From 163 to 279 mg/kg	[131]
	Brazil	-	113.5 ± 16.9 mg/kg	[139]
	Argentina	-	From 34.0 to 105.0 mg/kg	[132]
	Chile	-	From 5.5 to 105.0 mg/kg	[135]
	Spain	-	From 17.4 to 460.7 mg/kg	[135]
	Poland	from May to September 2018	40.1 ± 2.7 mg/kg	[120]
	Lithuania	from May to September 2018	From 31.9 to 102.1 mg/kg	[120]
	Brazil	from January to March 2011	From 10 to 50 mg/kg	[134]
	Serbia	2013	From 19.2 to 241 mg/kg	[140]
	Greece	Between spring 2013 and August 2014	From 30.7 to 383.8 mg/kg	[137]
Honey	Morocco	Summer of 2015 and 2016	From 1.09 to 4.02 mg/kg	[145]
	Lithuania	2018	From 1.08 to 5.15 mg/kg	[120]
	Italy	2018	2.03 mg/kg	[120]
	Greece	2018	2.18 mg/kg	[120]
	Serbia	2016	From 0.78 to 1.84 mg/kg	[153]
	New Zealand	Autumn 2007	From 0.2 to 2.46 mg/kg	[155]
	Malaysia	From January 2013 to March 2014	From 1.25 to 4.56 mg/kg	[144]
	Brazil	From July 2007 to March 2009	From 0.07 to 1.85 mg/kg	[146]
	Palestine	Between April and August 2009	From 2.06 to 8.36 mg/kg	[154]
	Malaysia	between July 2010 and August 2011	From 4.70 to 173.77 mg/kg	[162]
	China	-	From 0.59 to 22.85 mg/kg	[152]
	Poland	2004–2005	From 0.30 to 8.40 mg/kg	[143]
	Egypt	Cotton season 2011	From 1.63 to 2.57 mg/kg	[142]
	Ethiopia	-	From 0.370 to 1.124 mg/kg	[150]
	Kenya	-	From 0.05 to 0.35 mg/kg	[147,151]
	Cuba	-	From 0.20 to 1.71 mg/kg	[141]
	Mexico	--	From 1.51 to 6.80 mg/kg	[148]
Royal jelly	Germany,	2017	From 18.1 to 19.7 mg/kg	[120]
	Lithuania	2018	24.1 mg/kg	[120]
	France and china	May, June, and July 2001	From 19.4 to 24.8 mg/kg	[156]
	China	Between September and October 2014	From 17.56 to 24.91 mg/kg	[157]
	China	-	From 20 to 26 mg/kg	[158]

#### 6.1.2. Role of Zinc as Adjuvant Therapy in the Management of COVID-19: A Database of Clinical Trials

As reported in Table 2, zinc has been the subject of numerous clinical trial studies. Data were incomplete, and in most clinical investigations the results are not available or are still pending. Functional micronutrients are essential elements for the proper functioning of the immune system and play crucial roles in supporting health and nutritional well-being. To date, the Food Safety Authority (FSA) has authorized and recommended several health claims referring to the involvement of certain critical nutrients (vitamins B6, B9, B12, A, D, C, and Cu, Fe, Se) in the proper functioning of the immune system. Vitamins C D, E; Zn, and Se have been widely investigated as a promising way to boost the immune system and fight COVID-19 disease [163]. The database of clinical trials on zinc integration in COVID-19 treatment has been analyzed to better understand the benefit and drawbacks of various therapy regimes. As of 10 February 2022, 7479 clinical research studies have been registered in 220 countries, in which 69 studies have investigated the involvement of Zn against COVID-19 infection (available online at https://clinicaltrials.gov/ct2/home, accessed on 10 February 2022). Numerous preventive clinical studies have been designed to evaluate the prophylactic effect of zinc associated with hydroxychloroquine, ivermectin, and dietary supplement (vitamin A, B12, C, D3; copper and selenium). Among these studies, 3 studies have evaluated the combination of zinc and other functional micronutrients supplement (Table 2). The NCT04551339 trial focused on the association of vitamin E, vitamin C, copper, and beta-carotene in boosting the immune health to fight the COVID-19 pandemic. Beta-carotene, vitamin E, and vitamin C are known as antioxidant components with beneficial immunomodulatory actions [164,165]. In addition to zinc, these micronutrients modulate the immune response and prevent COVID-19 complications through one or multiple mechanisms of action. For instance, vitamin C, a water-soluble vitamin contains in many functional foods including beehive products, plays a crucial role as a co-factor and a modulator of numerous immune system signaling pathways. In a randomized clinical trial study, Bing et al. [166] reported that 56 hospitalized COVID-19 pneumonia patients treated with a high dose of vitamin C, 12 g/50 mL every 12 h for 7 days, 12 mL/h expressed lower levels in IL-6, and a rise in the PaO_2_/FiO_2_ (229 vs. 151 mmHg) as well as significantly improving the intensive care unit (IUC) mortality of severe patients with organ failure assessment score, SOFA score ≥ 3.

The combination of micro-antioxidant nutrients as a safe and economic intervention in the treatment/management of COVID-19 has already been documented. In a recent study, Atousa et al. [167] reported that a daily administration of vitamin C (1000 mg), vitamin E (400 IU), and the national treatment regime (hydroxychloroquine) to the adults hospitalized COVID-19 patients during the hospitalization period until hospital discharge or intensive care unit (ICU) improved the clinical signs and decreases the severity of the disease. However, this finding did not reach statistical significance. Furthermore, the NCT04468139 trial combined quercetin (500 mg), bromelain (500 mg), vitamin C (1000 mg), and zinc (50 mg) as quadruple therapy for COVID-19 patients. Quercetin, a flavonol component highly present in agro-food byproducts, has a long history as a powerful antioxidant molecule in preventing and combating the oxidative damages of several pro-oxidant agents [168]. More importantly, previous clinical trials (NCT04578158, NCT04377789, NCT04861298, and NCT04861298) have shown the importance of quercetin supplementation as a preventive or a therapeutic tool for COVID-19 treatment. 

The use of zinc as an active supplement or/and adjuvant treatment was recently adopted as a management strategy of the COVID-19 outbreak and its serious complications. NCT04446104, NCT04384458, and NCT04395768 are preventive clinical trials, evaluating ivermectin, hydroxychloroquine, and/or vitamin supplements in combination with zinc as a prophylactic approach for healthcare workers at high-risk and the general public. In addition to anti malarian action, hydroxychloroquine has been documented as having various clinical effects including anti-autophagy, anti-inflammatory, and immunosuppressive activities [169]. Recently, both chloroquine and hydroxychloroquine have shown promising potential in clinical setups to fight COVID-19 and its related complications. Owing to these properties and taking into consideration the multifunctioning role of zin in the human body, the combination of zinc, antioxidant nutrients, and hydroxychloroquine has been promoted as a COVID-19 treatment and prevention medication [170,171]. Interestingly, hydroxychloroquine/chloroquine act as zinc ionophores, increasing the intracellular concentration of zinc and thus, enhancing the suppression of SARS-CoV-2 RNA-dependent-RNA polymerase [172,173]. NCT00944359, NCT04482686, and NCT04447534 trials aim to discover a better COVID-19 therapy option by combining antiviral drugs/dietary supplements with zinc. In a retrospective observational study, the combination of zinc sulfate with hydroxychloroquine and azithromycin act synergistically and significantly decrease the mortality, the likelihood of being discharge to a home, as well as reducing the need for invasive ventilation or an intensive care unit of COVID-19 patients when compared to the conventional therapy (hydroxychloroquine alone or associated with azithromycin) [174]. Moreover, a 2-center, retrospective study on critically ill COVID-19 patients documented that the incorporation of zinc as adjunctive therapy was associated with a lower odds of acute kidney injury development during ICU stay as a serious complication of SARS-CoV-2 infection [175]. On the contrary, Szarpak et al. [176] have shown that zinc supplementation did not have any beneficial impact on ICU lengths stay.

The common denominator among these clinical trials is the incorporation of zinc as a valuable micronutrient either in the prevention or treatment of COVID-19 in virtue of its antiviral, anti-inflammatory and antioxidant properties [177,178]. Based on its anti-inflammatory properties, zinc improves the severity of SARS-CoV-2 by down-regulating the NF-κB signaling pathway, a key stimulator of pro-inflammatory cytokines, and thus, boost the IFN-mediated antiviral effects [179]. 

Acting synergistically, several bioactive molecules, especially phenolic components, antioxidant nutrients, and some anti-viral drugs may be effective in reducing COVID-19 symptoms or preventing its progression. 

### 6.2. Polyphenols

#### 6.2.1. Phenolic Compounds in Bee Products

Polyphenols or phenolic compounds are a heterogeneous class of chemical compounds belonging to secondary metabolites of plants; they are composed mainly of phenolic acids, and flavonoids (flavonols, flavones, flavanols, flavanones, anthocyanidin, chalcones, and isoflavones) [80,180,181]. 

The phenolic composition of bee products mainly depends on their floral origin [182]. The quantification of phenolic compounds in bee products from some countries is presented in Table 3. It was found that the most common polyphenols that existed in honey are flavonoids and acid phenolic compounds. The flavonoids include apigenin, catechin, chrysin, galangin, genistein, isorhamnetin, kaempferol, luteolin, myricetin, pinobanksin, pinocembrin, quercetin, and rutin. The common phenolic acids in honey are 2-cis,4-trans abscisic acid, 2-hydroxycinnamic acid, caffeic acid, chlorogenic acid, cinnamic acid, ellagic acid, ferulic acid, gallic acid, *p*-coumaric acid, *p*-hydroxybenzoic acid, protocatechuic acid, sinapic acid, syringic acid, and vanillic acid [80]. Whereas in propolis, the phenolic compounds were divided into phenolic acids derivatives, flavones, flavonols, flavanones, and dihydroflavonols. Additionally, Brazilian propolis is characterized by the presence of chrysin, pinocembrin, galangin, caffeic acid phenylethyl ester (CAPE), pinobanksin-3-o-acetate, and artepillin C [183].

Phenolic compounds that existed in bee pollen are as follows: gallic acid, protocatechuic acid, caffeic acid, ferulic acid, chlorogenic acid, para-coumaric acid, ortho-coumaric acid, luteolin, apigenin, chrysin, quercetin, rutin (Q 3-o-rutoside), kaempferol, myricetin, galangin, naringenin, pinocembrin, genistein [184]. Concerning bee venom, many biological properties including antiviral and antioxidant activities are attributed to melittin, a major peptide component ofbeevenom [185,186].

**Table 3:** Phenolic compounds in bee products.

Bee Products	Country	Collection Period	Content in Individual Polyphenols (mg/kg)	Reference
Propolis	Morocco	From the first of May to mid-June 2018	Catechin (9.6 ± 1.17); vanilic acid (5.6 ± 0.90); *p*-coumaric acid + epicatechin (ranged from 5.4 ± 0.21 to 190.5 ± 42.00); *o*-coumaric acid (ranged from 4.1 ± 0.10 to 180.2 ± 0.54); ferulic acid (ranged from 8.3 ± 0.02 to 31.0 ± 4.05); ellagic acid (ranged from 6.5 ± 0.19 to 134.8 ± 19.35); naringin (ranged between 7.6 ± 0.89 and 68.10 ± 7.81); hesperidin (ranged between 7.2 ± 1.28 and 67.2 ± 6.26); apigenin (ranged between 7.1 ± 0.12 and 262.8 ± 40.03); cinnamic acid (ranged between 0.9 ± 0.07 and 34.2 ± 3.54); resveratrol (ranged between 9.9 ± 0.04 and 38.7 ± 9.24); rosmarinic acid (ranged between 13.0 ± 0.12 and 65.6 ± 14.72); rutin (ranged between 3.7 ± 0.88 and 160.6 ± 03.85); chlorogenic acid (ranged between 8.8 ± 0.01 and 32.0 ± 7.23); quercetin (ranged between 7.1 ± 0.19 and 26.7 ± 0.05); kaempferol (ranged between 5.1 ± 0.94 and 303.2 ± 21.15)	[55]
	Italy	Not mentioned	Chrysin (781.5 ± 80.1); Apigenin (132.1 ± 21.6); Acacetin (1133.3 ± 256.2); Tectochrysin (130.9 ± 21.6); Pinocembrin (769.4 ± 77.9); Pinostrobin (575.6 ± 48.6); Sakuranetin (152.9 ± 20.6); Galangin (70.4 ± 11.8); Kaempferide (39.8 ± 3.6); Quercetin (153.5 ± 18.6); Prenyl caffeate (33.3 ± 2.6); Benzyl caffeate (527.1 ± 61.8); caffeic acid phenylethyl ester (1745.2 ± 245.2)	[187]
	China	Not mentioned	Chrysin (2333.0 ± 350.0); Apigenin (178.6 ± 34.8); Acacetin (1578.0 ± 141.9); Tectochrysin (238.3 ± 24.6); Pinocembrin (2087.5 ± 347.0); Sakuranetin (146.0 ± 18.4); Galangin (1400.3 ± 126.6); Kaempferide (38.8 ± 5.0); Quercetin (70.9 ± 9.7); Prenyl caffeate (56.2 ± 6.0); Benzyl caffeate (1477.0 ± 116.6); caffeic acid phenylethyl ester (2525.0 ± 199.3)	[187]
	Argentina	Not mentioned	Chrysin (2347.6 ± 215.7); Apigenin (336.9 ± 41.7); Acacetin (831.5 ± 16.0); Tectochrysin (224.7 ± 29.0); Pinocembrin (3362.5 ± 418.8); Sakuranetin (27.5 ± 4.1); Galangin (2253.7 ± 294.4); Kaempferide (38.1 ± 4.6); Prenyl caffeate (29.7 ± 4.2); Benzyl caffeate (1180.1 ± 200.8); caffeic acid phenylethyl ester (1111.6 ± 125.6)	[187]
	Ukraine	Not mentioned	Chrysin (922.0 ± 111.2); Apigenin (177.4 ± 15.2); Acacetin (658.9 ± 67.0); Tectochrysin (153.6 ± 18.7); Pinocembrin (1196.5 ± 91.9); Pinostrobin (1479.3 ± 303.5); Sakuranetin (2184.0 ± 196.1); Galangin (952.9 ± 106.8); Kaempferide (91.9 ± 13.7); Quercetin (28.9 ± 4.1); Prenyl caffeate (53.9 ± 6.1); Benzyl caffeate (465.1 ± 31.9); caffeic acid phenylethyl ester (1145.9 ± 98.6)	[187]
	Macedonia	Not mentioned	Chrysin (1649.8 ± 177.5); Apigenin (236.4 ± 34.1); Acacetin (1343.0 ± 200.2); Tectochrysin (987.1 ± 104.0); Pinocembrin (2112.0 ± 184.5); Pinostrobin (3816.0 ± 397.1); Sakuranetin (2203.4 ± 269.0); Galangin (903.9 ± 88.2); Kaempferide (43.2 ± 7.0); Quercetin (94.8 ± 10.3); Benzyl caffeate (400.6 ± 62.6); caffeic acid phenylethyl ester (1263.1 ± 212.6)	[187]
	Morocco	May 2018	Ferrulic acid (40.60 ± 0.6); o-Coumaric acid (35.47 ± 0.2); Chlorogenic acid (25.31 ± 0.0); Rosmarinic acid (222.02 ± 6.2); Vanilic acid (10.58 ± 0.1); Ellagic acid (37.94 ± 0.1); Catechin (18.83 ± 0.1); Naringin (290.19 ± 0.2); Hesperidin (271.77 ± 0.0); Quercetin (14.78 ± 0.2); Apigenin (50.37 ± 0.8); Kaempferol (26.48 ± 1.2); Rutin (34.37 ± 1.3); Resveratrol (86.25 ± 0.2)	[54]
	Morocco	July 2018	Vanillic acid (8.61 ± 0.30); o-Coumaric acid (11.44 ± 4.63); Ferulic acid (18.84 ± 0.21); Ellagic acid (28.55 ± 1.99); Naringin (35.78 ± 4.10); Hesperidin (417.18 ± 50.0); Apigenin (38.39 ± 2.60); Resveratrol (116.89 ± 12.7); Rosmarinic acid (470.35 ± 52.00); Rutin (12.40 ± 0.42); Chlorogenic acid (16.11 ± 0.12); Quercetin (12.02 ± 0.13); Kaempferol (21.90 ± 1.60)	[56]
Bee bread	Morocco	Not mentioned	Kaempferol- *O*-hexosyl-*O*-rutinoside (570 ± 10); Quercetin-*O*-hexosyl-*O*-hexoside (950 ± 30); Methylherbacetrin-*O*-dihexoside (545 ± 4); isorhamnetin-*O*-hexosyl-*O*-rutinoside (1480 ± 50); Quercetin-*O*-pentosyl-hexoside (580 ± 10); Quercetin 3-*O*-rutinoside (530 ± 10); methylherbacetrin-3-*O*-rutinoside (510 ± 10); isorhamnetin-*O*-pentosyl-hexoside (930 ± 10); kaempferol-3-*O*-rutinoside (510 ± 10); isorhamnetin-*O*-rhamnoside-hexoside (560 ± 10)	[117]
	Romania	spring of 2020	kaempferol (31.25), myricetin (3.15), luteolin (1.17), rosmarinic acid (0.23), Caffeic acid (0.10), *p*-Coumaric acid (0.11), Quercetin (0.06)	[188]
	North-East European countries	2015	Gallic Acid (300); Caffeic Acid (between 700 to 6400); Catechin (between 900 and 52,100); Clorogenic acid (between 800 to 1400); *p*-Coumaric acid (between 2300 and 11,400); Ferulic acid (between 400 and 1100); Naringenin +Quercetin (between 400 and 3200): Apigenin +Kaempferol (between 600 and 15,800); Pinocembrin (between 400 and 56,600); CAPE +Galangin (between 2400 and 71,900)	[189]
	South-West European countries	2015	Caffeic acid (between 4600 to 6600); Catechin (between 1700 and 6300); Clorogenic acid (between 400 to 7300); *p*-Coumaric acid (between 2000 and 12,200); Ferulic acid (between 800 and 2900); Naringenin +Quercetin (between 300 and 1000); Apigenin +Kaempferol (between 4000 and 32,200); Pinocembrin (between 13700 and 33,900); CAPE +Galangin (between 1300 and 110,600)	[189]
	South American tropical zone	2015	Gallic acid (300); Catechin (between 20800 to 34,100); Clorogenic acid (200); *p*-Coumaric acid (between 1200 and 2800); Ferulic acid (between 300 and 800); Naringenin +Quercetin (between 500 and 700); Apigenin +Kaempferol (between 1500 and 6500); Pinocembrin (33,300); CAPE +Galangin (between 7600 and 9000)	[189]
Bee pollen	Morocco	May 2018	Ferrulic acid (17.17 ± 0.4); cinnamic acid (46.01 ± 7.8); o-Coumaric acid (27.10 ± 1.9); Rosmarinic acid (127.30 ± 6.2); Gallic acid (32.54 ± 2.2); Vanilic acid (6.13 ± 0.1); Ellagic acid (13.02 ± 0.0); Naringin (113.71 ± 6.8); Hesperidin (15.63 ± 6.8); Quercetin (48.12 ± 2.8); Apigenin (162.85 ± 17.7); Rutin (95.36 ± 3.7); Resveratrol (44.00 ± 0.4)	[54]
	Turkey	2007–2008	Gallic acid (from 9.46 to 18.59); Protocatechuic acid (from 4.73 to 19.77); *p*-OH benzoic acid (from 2.74 to 122.68); Chlorogenic acid (from 14.64 to 75.08); Vanillic acid (from 22.96 to 87.02); Caffeic acid (from 10.88 to 98.03); Syringic acid (from 10.55 to 259.53); Epicatechin (from 39.15 to 520.02); *p*-Coumaric acid (from 34.16 to 127.85); Ferulic acid (from 36.83 to 230.55); Benzoic acid (from 46.87 to 1077.64); Rutin (from 25.59 to 692.85); *o*-Coumaric acid (from 2.63 to 42.23); Abscisic acid (from 21.04 to 288.70); tert-Cinnamic acid (from 6.82 to 56.38); Quercetin (from 61.40 to 499.20)	[190]
	Chile	Not mentioned	Feluric acid (7.75 ± 0.39); Syringic acid (7.92 ± 0.40); *p*-coumaric acid (52.17 ± 2.60); Rutin (86.60 ± 4.30); Luteolin (28.37 ± 1.42); Cinnamic acid (5.77 ± 0.29); Quercetin (32.72 ± 1.64); Miricetin (6.76 ± 0.34)	[191]
Honey	Morocco	July 2018	Vanillic acid (2.90 ± 0.01); Ferulic acid (8.35 ± 0.01); Ellagic acid (5.09 ± 0.02); Cinnamic acid (4.25 ± 0.01); Chlorogenic acid (7.06 ± 0.11); Gallic acid (30.06 ± 0.23)	[56]
	Chile	Not mentioned	Chlorogenic acid+caffeic acid (19.67 ± 0.98); Sinapic acid (from 17.58 to 23.86); Feluric acid (from 4.21 to 16.29); Syringic acid (4.73 ± 0.24); Luteolin (from 8.16 to 11.55); Cinnamic acid (3.00 ± 0.15); Quercetin (5.88 ± 0.29); Kaempherol (from 17.00 to 37.00)	[191]
	Hong Kong, Spain, Italy, Korea, China, Canada, Brazil, New Zealand, and Germany	Not mentioned	gallic acid (from 20 to 66); protocatechuic acid (from 7.1 to 63); 2,3,4-trihydroxybenzoic acid (from 11 to 28); protocatechualdehyde (from 7 to 63); *p*-hydroxybenzoic acid (from 8.1 to 21.7); vanillic acid (5.1); vanillin (from 4.1 to 22.7); syringic acid (from 1.1 to 87.7); caffeic acid (from 6.61 to 23.6); chlorogenic acid (from 11.4 to 21.2); *p*-coumaric acid (from 8.7 to 13.6); syringaldehyde (from 1.7 to 87.7); genistic acid (from 8.2 to 39.6)	[192]
	Morocco	Not mentioned	Methyl syringate (from 277.93 to 443.89); Epicatechin (from 30.67 to 179.29); Syringic acid (from 25.16 to 105.86); Catechin (from 14.56 to 66.13); 4-coumaric acid (from 5.13 to 49.43); Gallic acid (from 0.00 to 34.40); Quercetin (from 7.53 to 42.77); Apigenin (from 1.25 to 33.38); Luteolin (from 0.00 to 22.08); Kaempferol (from 5.24 to 19.64); Naringenin (from 0.00 to 42.41); Formononetin (from 0.87 to 17.88); Genistein (from 0.00 to 24.00); 3-coumaric acid (from 2.40 to 8.66); Daidzein + Pelargonidin (from 1.84 to 8.16); 2-coumaric acid (from 0.45 to 8.17); Biochanin A (from 0.86 to 4.10); Cyanidin (from 0.00 to 2.30)	[193]

#### 6.2.2. Role of Polyphenols in the Management of COVID-19

**Figure 3:**
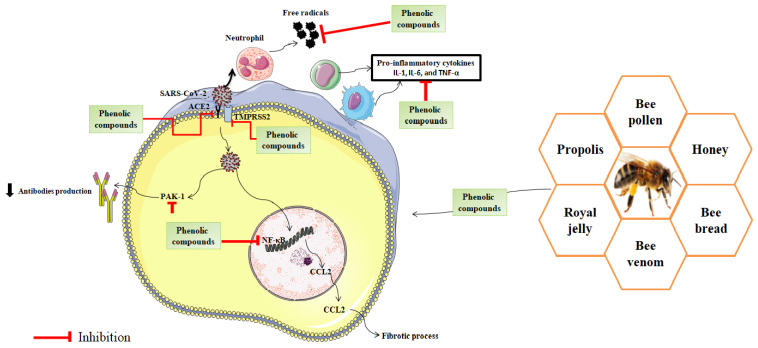
Possible mechanism of action of phenolic compounds in the management of COVID-19.

Inflammation is a major problem caused after the binding of SARS-CoV-2 to the ACE2 receptor of the host cell through spike protein and the activation of transmembrane protease serine 2 (TMPRSS2). After viral endocytosis, RAC/CDC42-activated kinase 1 (PAK1) and chemokine (C-C motif) ligand 2 (CCL2) were stimulated, reducing the production of antibodies and inducing a fibrotic process. These intracellular signals lead to the activation of nuclear transcription factor κB(NF-κB)which causes local inflammation, tissue damage, and the appearance of complications (Figure 3) [194].

Meanwhile, there is evidence that viral infections are associated with an increase in reactive oxygen species (ROS) levels and a decrease in antioxidant defenses (Figure 3). Exposure to SARS-CoV-2 leads to the inhibition of NRF2-mediated pathways and neutrophilia, which causes deleterious effects on pulmonary cells leading to hypoxic respiratory failure and the complications observed in severe COVID-19 cases [195]. Furthermore, oxidative stress leads to protein oxidation, lipid peroxidation, and DNA damage, these damages induce the release of inflammatory signals [196]. 

Polyphenols are natural compounds well known for their antioxidant and anti-inflammatory effects. The antioxidant activity of polyphenols is related to their capacity to react with ROS using different reaction pathways such as the inhibition of the enzymes responsible for ROS production, the up-regulation of endogenous antioxidant defenses, or scavenging reactive oxygen species [197]. 

It seems that the main pathways, by which polyphenols-rich bee products can attenuate SARS-CoV-2 infection, are the modulation of the immune system, the prevention of inflammatory responses, and the reduction in oxidative stress (Figure 3). It was reported that phenolic compounds modulate enzymatic and signaling pathways involved in the inflammation. For instance, it was reported that quercetin is an effective inhibitor of NF-κB, TNF-α, NO, and PLA_2_ pathways. Resveratrol exerted an anti-inflammatory effect by inhibiting NF-κB and TPA-induced COX-2 expression. Similarly, caffeic acid phenethyl ester (CAPE) inhibits inflammatory reaction via the reduction of NF-κB [198].

Recently, numerous published reports revealed that phenolic compounds found in bee products can react with SARS-CoV-2 and blocks its entry into human cells (Figure 3 and Table 4). It was found in a study published by Sahlan et al. that glyasperin A and broussoflavonol F, Sulawesi propolis compounds, have favorable interaction profiles with SARS-CoV-2 main protease (PDB ID 6Y2F) catalytic sites (His41 and Cys145) with binding similarities of 75% and 63%, respectively, compared to potent inhibitors [199]. Similarly, caffeic acid phenethyl ester can bind to the substrate-binding pocket of SARS-CoV-2 M^pro^ with efficacy and binding energies equivalent to an already claimed N3 protease inhibitor [200].

In addition, the molecular docking of ten flavonoid compounds: apigenin, chrysin, fisetin, galangin, hesperitin, luteolin, morin, naringin, quercetin, revealed that all of the flavonoids studied have a high binding affinity with the active site of the spike protein of SARS-CoV-2 [201].

The antiviral effect of 6 compounds present in honey and propolis (3-phenyllactic acid, caffeic acid phenethyl ester, caffeic acid, chrysin, galangin, lumichrome) was studied in silico. The results showed that four compounds (caffeic acid phenethyl ester, caffeic acid, chrysin, galangin) had a strong binding affinity with a good glide score and may inhibit the COVID-19 M^pro^ and replication of the virus [202].

In a study conducted by Vijayakumar et al. [203], the molecular docking of natural flavonoids against essential proteins of SARS-CoV-2 (RNA dependent RNA polymerase (rdrp), main protease (M^pro^) and Spike (S) protein) revealed that cyanidin may suppress rdrp by binding at asp761 catalytic residue and halting the viral replication process. In addition, the following flavonoids can interact on the spike proteins’ key rbd and inhibit the spread to receptors and thus limit viral spread: daidzein, eriodictyol, fisetin, genistein, kaempferol, myricetin, quercetin, arbutin, chalconaringenin, phloretin, and liquiritin. 

A total of 220 phenolic compounds were tested in a study published by Pitsillou et al. The results of in silico and in vitro using an enzymatic inhibition assay indicated that hypericin, rutin, and cyanidin-3-*O*-glucoside can be considered lead compounds in the inhibition of SARS-CoV-2 papain-like protease (PL^pro^) [204].

In the same vein, docking studies of rutin, caffeic acid phenethyl ester, quercetin, kaempferol, pinocembrin, pinobanksin, galangin, chrysin, *p*-cumaric acid, and benzoic acid revealed that rutin and caffeic acid phenethyl ester have the highest affinity to COVID-19 3CL-protease and S1 Spike [69] (Table 4).

Interestingly, several clinical trials have been conducted to test the beneficial effect of polyphenols against SARS-CoV-2 infection. For instance, the NCT04666753 trial aims to describe the effect of the immunomodulation treatment (60 mg of zinc orotate, 96 μg of selenium, 20,000 IU of cholecalciferol, 300 mg of ascorbic acid, 480 mg of ferulic acid, 90 mg of resveratrol on COVID-19 patients (adults and older adults). Antioxidant compounds and other food micronutrients with valuable additional functionalities are important in resolving other COVID-19-related issues. For instance, human trial studies documented that ferulic acid and resveratrol have strong antidiabetic, cardioprotective, and renoprotective effects [205,206]. Thus, these components could be proposed as adjuvant active molecules and novel dietary approaches for the management of COVID-19 complications.

Furthermore, as of 10 February 2022, quercetin has been approved in 14 clinical trials for profylaxis and managing COVID-19 symptoms (available online at https://clinicaltrials.gov/ct2/home, accessed on 10 February 2022). For instance, the NCT04578158 trial, a randomized, open-labeled, and controlled study, investigates the adjuvant benefits of quercetin Phytosome (400 mg/day, orally for 30 days) in community-based subjects with confirmed SARS-CoV-2 infection. In addition to quercetin, resveratrol, a stilbenoid that highly presents in bee products, has been the object of 4 clinical trials (NCT04400890, NCT04799743, NCT04542993, and NCT04666753 trials). The NCT04400890 trial aims to evaluate the safety and explore the effectiveness of resveratrol treatment (1000 mg, 4 times a day, for 15 days) against COVID-19 infection. Published data support that resveratrol inhibits coronavirus replication through the down-regulation of mRNA and nucleocapsid protein expressions of SARS-CoV-2 [207,208].

## 7. Conclusions

In the absence of specific antiviral drugs against SARS-CoV-2, natural remedies such as apitherapy could alleviate the complications associated with COVID-19. Considering the crucial role of the immune system in fighting SARS-CoV-2 infection, boosting immunity was highly recommended. In this direction, the present review highlights and provides an overview of zinc and phenolic compounds found in bee products and their direct and indirect actions on the immune system in fighting against this emergent public health crisis. The problems which arise are that the nature, quality, and quantity of these bioactive molecules in bee products differ from one country to another. To overcome this major drawback, an international norm, ISO/TC 34/SC 19, is currently in progress to standardize the whole process and circulation of bee products.

## Figures and Tables

**Table 2:** Zinc as adjuvant therapy in the management of COVID-19: clinical trials.

Clinical Trial Study	Dose/Route and Treatment Duration	Drugs/Dietary Supplement	Number of Participants/Age/Sex	ClinicalTrials.gov Identifier/Phase
Efficacy of subcutaneous ivermectin with or without zinc in COVID-19 patients	20 mg/day of zinc sulfate, orally 3 times a day	Ivermectin and zinc	180/18 years and older/males and females	NCT04472585/Phase II
A randomized, placebo-controlled study evaluating the efficacy of zinc for the treatment of COVID-19 in the outpatient setting	220 mg/day of zinc Sulfate, once daily, orally for 5 days	Zinc sulfate	300/18 years and older/males and females	NCT04621461/Phase IV
A study of quintuple therapy to treat COVID-19 infection Hydroxychloroquine	No available data	Hydroxychloroquine, Azithromycin/Vitamin C, vitamin D and zinc	600/18 years and older/both sexes	NCT04334512/Phase II
Zinc versus multivitamin micronutrient supplementation to support immune health in the setting of COVID-19 pandemic: a randomized study	11 mg/day of zinc, orally during 3 months	Vitamin E, vitamin C, zinc, copper and beta-carotene	2700/between 18 to 90 years/both sexes	NCT04551339/Complited
Anti-inflammatory/antioxidant oral nutrition supplementation in COVID-19	Oraldietary supplement containing 5.7 mg zinc for 14 days	Supplement enriched with zinc, selenium vitamin A	30/between 18 to 65 years/both sexes	NCT04323228/Phase III
The study of quadruple therapy zinc, quercetin, bromelain, and vitamin C on the clinical outcomes of patients infected with COVID-19	50 mg/day, orally during 10 days	Quercetin, bromelain, vitamin C and Zinc	60/18 years and older adult)/males and females	NCT04468139/Phase IV
A preventive treatment for migrant workers at high-risk of COVID-19	80 mg/day of zinc tablet, once a day for 42 days	Hydroxychloroquine, Ivermectin, povidone-iodine/vitamin C, and zinc	5000/between 21 to 60 years/both sexes	NCT04446104/Phase III
Comparative study of hydroxychloroquine associated with zinc and ivermectin in COVID-19 prophylaxis	20 mg/day of zinc twice a day for 45 days	Hydroxychloroquine and Ivermectin/zinc	400/between 18 to 70 years/both sexes	NCT04384458/not available
A study of hydroxychloroquine and zinc in the prevention of COVID-19 infection in military healthcare workers	15 mg/day of zinc sulfate, orally during 2 months	Hydroxychloroquine/zinc	660/between 18 to 65 years/both sexes	NCT04377646/Phase III
International ALLIANCE study of therapies to prevent progression of COVID-19	30 mg/day of zinc citrate, orally for 14 days	Hydroxychloroquine and Azithromycin/Zinc Citrate, Vitamin C, vitamin D3, and vitamin B12	200/18 years and older/both sexes	NCT04395768/Phase II
Community-based intervention trial to compare theimpact of preventive and therapeutic zinc supplementation programs amoung young children	20 mg/day of zinc, orally for 10 days during episodes of diarrhea	Zinc	7680/18 years and older/both sexes	NCT00944359/not available
Hydroxychloroquine and zinc with either azithromycin or doxycycline for treatment of COVID-19 in outpatient setting	220 mg of zinc sulfate once a day, orally for 5 days	Hydroxychloroquine, Azithromycin, Doxycycline/zinc	750/30 years and older/males and females	NCT04370782/Phase IV
Trial of combination therapy to treat COVID-19 infection	No available data	Ivermectin, doxycycline Hcl/zinc, vitamin C, and vitamin D	31/between 18 to 75 years/males and females	NCT04482686/Pahse I
Does zinc supplementation enhance the clinical efficacy of chloroquine/hydroxychloroquine in treatment of COVID-19?	No available data	Chloroquine/zinc	200/adults/males and females	NCT04447534/Phase III

**Table 4:** Efficacy of flavonoids in vitro and in silico against SARS-CoV-2.

Type of the Study	Compounds Used	Key Results	Reference
In silico	glyasperin A and broussoflavonol F	Both compounds have favorable interaction profiles with SARS-CoV-2 main protease (PDB ID 6Y2F) catalytic sites (His41 and Cys145) with binding similarities of 75% and 63%, respectively, compared to potent inhibitors.	[199]
In silico	caffeic acid phenethyl ester	Caffeic acid phenethyl ester can bind to the substrate-binding pocket of SARS-CoV-2 M^pro^with efficacy and binding energies equivalent to an already claimed N3 protease inhibitor	[200]
In silico	apigenin, chrysin, fisetin, galangin, hesperitin, luteolin, morin, naringin, quercetin, rutin, qercetin, kaempferol, *p*-coumaric acid, chrysin, luteolin, ribavirin	All the flavonoids studied have a high binding affinity with the active site of the spike protein of SARS-CoV-2	[201]
In silico	3-phenyllactic acid, caffeic acid phenethyl ester, caffeic acid, chrysin, galangin, lumichrome	caffeic acid phenethyl ester, caffeic acid, chrysin, galanginhave a strong binding affinity with a good glide score and may inhibit the COVID-19 M^pro^ and replication of the virus.	[202]
In silico	luteolin, apigenin, tangeritin, kaempferol, quercetin, myricetin, fisetin, hesperitin, naringenin, eriodicytol, luquiritin, genistein, daidzein, callophyllolide, cyanidin, delphenidin, malvidin, pelargonidin, peonidzin, arbutin, pheloretin, chalconaringenin	cyanidin may suppress rdrp by binding at asp761 catalytic residue and halting the viral replication process. daidzein, eriodictyol, fisetin, genistein, kaempferol, myricetin, quercetin, arbutin, chalconaringenin, phloretin, and liquiritin interact on the spike proteins’ key rbd and may inhibit spread to receptors.	[203]
In silico and in vitro	a total of 220 phenolic compounds were tested in the study	In silico and in vitro results indicate that hypericin, rutin, and cyanidin-3-*O*-glucoside can inhibit SARS-CoV-2 papain-like protease (PL^pro^)	[204]
In silico	rutin, caffeic acid phenethyl ester, quercetin, kaempferol, pinocembrin, pinobanksin, galangin, chrysin, *p*-cumaric acid, and benzoic acid.	Docking studies revealed that Rutin and Caffeic acid phenethyl ester showed the highest affinity to both targets (COVID-19 3CL-protease and S1 Spike)	[69]

## Data Availability

Data are available upon request.
